# Osteo-inductive effect of piezoelectric stimulation from the poly(l-lactic acid) scaffolds

**DOI:** 10.1371/journal.pone.0299579

**Published:** 2024-02-27

**Authors:** Ritopa Das, Duong Le, Ho-Man Kan, Thinh T. Le, Jinyoung Park, Thanh D. Nguyen, Kevin W.-H. Lo

**Affiliations:** 1 Department of Biomedical Engineering, University of Connecticut, School of Engineering, Storrs, CT, United States of America; 2 National Institute of Biomedical Imaging and Bioengineering, National Institute of Health, Bethesda, MD, United States of America; 3 Department of Mechanical Engineering, University of Connecticut, School of Engineering, Storrs, CT, United States of America; 4 Vinmec Research Institute of Stem Cell and Gene Technology, Vinmec Health System, Hanoi, Vietnam, United States of America; 5 The Cato T. Laurencin Institute for Regenerative Engineering, University of Connecticut, Storrs, CT, United States of America; 6 Institute of Materials Science (IMS), University of Connecticut, School of Engineering, Storrs, CT, United States of America; 7 Department of Medicine, Division of Endocrinology, University of Connecticut Health Center, School of Medicine, Farmington, CT, United States of America; Universidade de Trás-os-Montes e Alto Douro: Universidade de Tras-os-Montes e Alto Douro, PORTUGAL

## Abstract

Piezoelectric biomaterials can generate piezoelectrical charges in response to mechanical activation. These generated charges can directly stimulate bone regeneration by triggering signaling pathway that is important for regulating osteogenesis of cells seeded on the materials. On the other hand, mechanical forces applied to the biomaterials play an important role in bone regeneration through the process called mechanotransduction. While mechanical force and electrical charges are both important contributing factors to bone tissue regeneration, they operate through different underlying mechanisms. The utilizations of piezoelectric biomaterials have been explored to serve as self-charged scaffolds which can promote stem cell differentiation and the formation of functional bone tissues. However, it is still not clear how mechanical activation and electrical charge act together on such a scaffold and which factors play more important role in the piezoelectric stimulation to induce osteogenesis. In our study, we found Poly(l-lactic acid) (PLLA)-based piezoelectric scaffolds with higher piezoelectric charges had a more pronounced osteoinductive effect than those with lower charges. This provided a new mechanistic insight that the observed osteoinductive effect of the piezoelectric PLLA scaffolds is likely due to the piezoelectric stimulation they provide, rather than mechanical stimulation alone. Our findings provide a crucial guide for the optimization of piezoelectric material design and usage.

## Introduction

Bone repair and regeneration can be improved by physical stimulations such as mechanical and electrical stimulate [1]. In fact, both mechanical and electrical stimulations have been shown to be effective in promoting bone healing clinically [2, 3]. Mechanical stimulation such as compression, tension, shear, or fluid flow induce bone regeneration through mechanotransduction [4]. This process involves the conversion of mechanical signals into biochemical signals that can stimulate osteogenesis of primary cells such as mesenchymal stem cells [5]. This is achieved by activating signal pathways and modulating the expression of genes related to bone regeneration [6]. In addition, when a physical force is applied to bone, the bone tissue will generate electrical charges (via the piezoelectric effect) and the charges generated can enhance bone growth and repair [7]. Indeed, electrical stimulation (ES) has been shown to exhibit profound effects on bone repair [8]. ES has been used in a variety of clinical applications, such as the treatment of non-union fractures, spinal fusion, and osteoporosis [9]. ES causes bone regeneration through a process called electrotaxis by which electrical fields direct the migration of cells (e.g., mesenchymal stem cells) to the injury sites [10]. In addition, ES promote bone healing by enhancing cellular activity and the production of proteins and other molecules involved in bone formation [3]. Piezoelectricity is naturally present in the human body though it has not been fully explored for regenerating tissue. Native tissues, such as bone and cartilage all exhibit piezoelectric behavior, wherein electrical activity can be generated due to mechanical deformation during movements. Synthetic biomaterials with piezoelectric properties that can generate electric charges during mechanical deformation and vice versa, can be employed to create self-powered electrical stimulators that can effectively stimulate bone repair [11, 12]. Specifically, piezoelectric charges generated on the surface of the piezoelectric materials have been proven to effectively stimulate stem cell proliferation, osteogenic differentiation, and remodeling both *in vitro* and *in vivo* [13, 14]. However, it is unclear whether the mechanical forces or the electric charges are primarily responsible for the observed effects on bone regeneration. There is also a knowledge gap on the mechanisms of how mechanical and electrical stimulation can act together in such a piezoelectric stimulation to induce osteogenesis.

Several piezoelectric materials have been investigated for various biomedical applications. For instance, PLLA (poly-L-lactic acid) and PVDF (polyvinylidene fluoride) are both commonly used piezoelectric biomaterials [15]. PVDF is a synthetic polymer that is biocompatible, non-degradable and has been used in applications such as actuators, sensors, and energy harvesting devices [16]. PLLA is a biocompatible and biodegradable material that has been widely used in medical applications, including regenerative engineering particularly for bone tissue regeneration [11, 17]. Their piezoelectric properties enable them to convert mechanical activation into electrical stimulation for the growth and development of bone tissue in the body [18, 19]. Studies have shown that piezoelectric PLLA can induce bone formation both in *vitro* and *in vivo* without the need of exogenous growth factors or small molecules [13, 20]. PLLA is also more beneficial than PVDF to serve as a tissue scaffold because the material can safely degrade inside the body over time which will facilitate the cellular infiltration and tissue remodeling.

**Fig 1:**
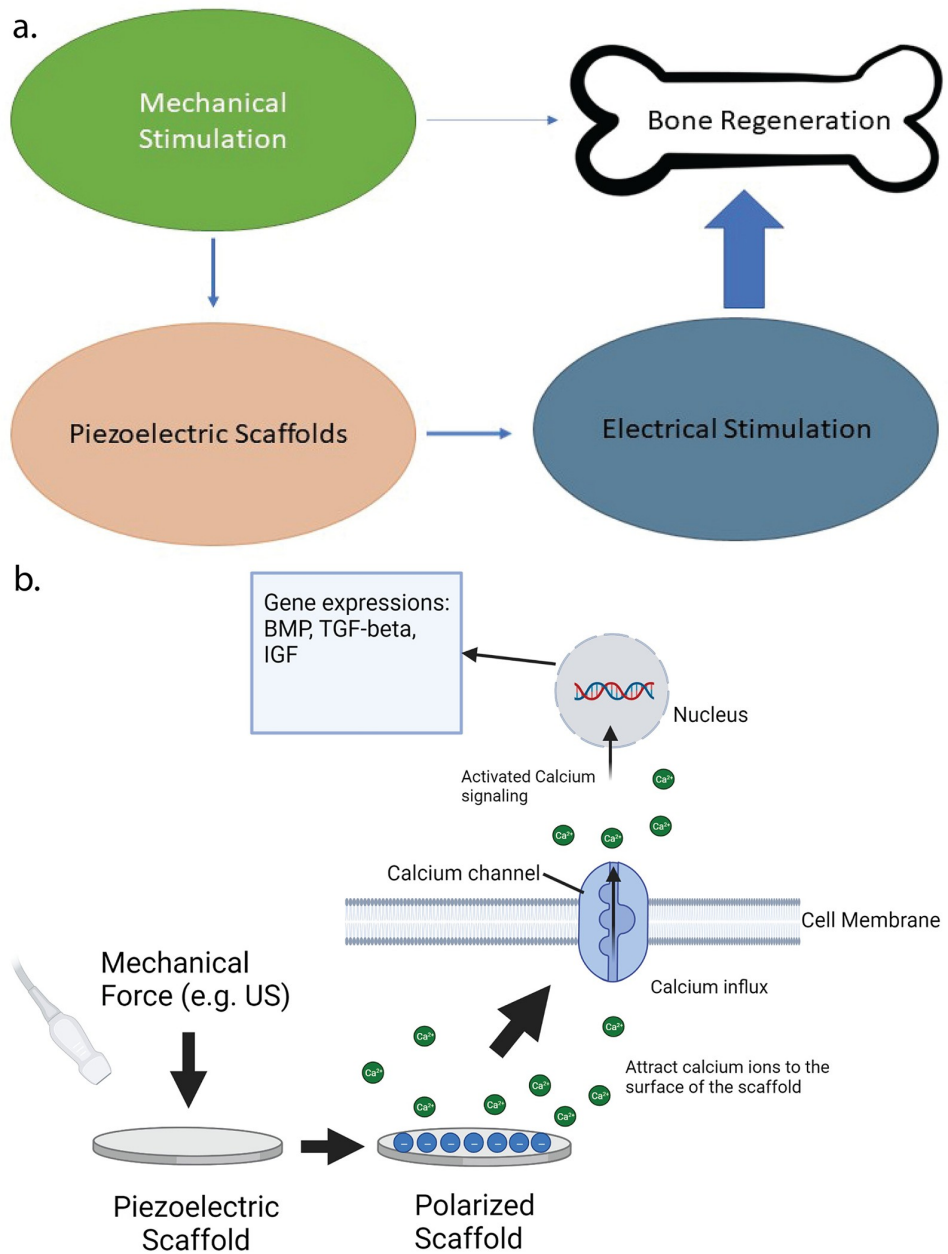
Schematic diagram of the bone regeneration mechanism of piezoelectric PLLA scaffolds. a. Piezoelectric stimulation involves a combination of electrical and mechanical stimulation. However, in the case of our scaffolds, the electrical stimulation plays a more important role than the mechanical stimulation. b. Piezoelectric charges induce intracellular calcium signaling in stem cells which upregulates production of BMP-2 and TGF-β and enhances osteogenesis.

In our previous works we have presented a piezoelectric PLLA nanofiber material which has superior piezoelectric properties [11, 21]. We have already demonstrated using this material as a bone scaffold that can increase osteogenic differentiation in Adipose derived stem cells in vitro and regeneration of calvarial bones in vivo [22]. However, the exact mechanism has still not been fully understood. Collectively, these observations prompted us to investigate the intrinsic osteogenic effects of piezoelectric PLLA biomaterials under mechanical activation. In this paper, we study the mechanism behind the osteogenic nature of this piezoelectric scaffold in order to understand how the piezoelectric charge van induce bone regeneration. We suggested that the osteoinductive effect of the piezoelectric PLLA scaffolds is likely due to piezoelectrical stimulation rather than mechanical stimulation alone (**Fig 1A**). We used ultrasound as our mechanical stimulation because it is simple to operate and apply and can stimulate the piezoelectric graft externally and non-invasively. Besides it is already widely used clinically. Our data showed that the intrinsic osteoinductivity of PLLA based piezoelectric stimulation is through the production and secretion of cell-based osteoinductive growth factors such as bone morphogenetic proteins (BMPs) and transforming growth factor-beta (TGF-β), which are triggered by calcium signaling pathways (**Fig 1B**). This study demonstrated that piezoelectric charges generated on the PLLA materials under mechanical activation of ultrasound (US) enhance the stem cell migration and trigger intracellular calcium oscillation and such a change may directly trigger stem cell osteo-differentiation and/or induce cytokine-based inductive autocrine and paracrine loops [23].

## Materials and methods

### Fabrication of PLLA nanofiber mat

The poly (L-lactic acid) (PLLA) nanofiber mat was prepared by electrospinning as per the protocol described in our previous work [21]. PLLA (PURASORB PL38) was obtained from Corbion Purac (Amsterdam, Netherlands). A solution of 0.8g of the PLLA was prepared in a 1:4 mixture of N, N–Dimethylformamide (DMF, anhydrous, ≥99.9%) and dichloromethane (DCM) respectively (v/v); these solvents were purchased from Millipore Sigma (Burlington, MA). 5ml of the solution was loaded into a 10 ml syringe which was mounted on a syringe pump (New Era Pump Systems, Inc., Farmingdale, NY). The pump was set up in such a way that the polymer solution would flow at 2 ml/hr through a flat-tipped 21-gauge needle (Jensen Global, Santa Barbara, CA) with 14kV (kilovolts) applied to it. This polarized solution was then deposited onto aluminum foil, which was wrapped on an aluminum drum, grounded and rotating at speeds from 300–4,000 rpm (rotations per minute). The humidity in the electrospinning setup was maintained at 30 ± 5% whereas the temperature was the same as the ambient. This produced a PLLA nanofiber mat on the aluminum foil with different fiber alignments and diameters, depending on the collector drum speed. These fibrous PLLA films were then put through a two-step annealing process. At first, they were annealed at 105°C for 10hr in an oven (Quincy Lab Inc., Chicago, IL) following which, the oven was shut off and allowed to slowly cool to room temperature. The films were then peeled off the aluminum foil and placed between two Teflon FEP sheets (American DURAFILM, Holliston, MA) and on top of a glass slide. The sandwiched mat was then annealed in an oven at 160.1°C for 10hr. Afterwards, the oven was turned off and allowed to cool to room temperature.

### Measurement of the piezoelectric output of the PLLA films under ultrasonic stimulation

The annealed PLLA films were cut at a 45° angle to the fiber direction films into dimensions of 1.27 cm x 1.27 cm to maximize the shear force under applied perpendicular pressure. Aluminum (Al) foil was used to create the electrodes that would snugly sandwich the PLLA mat. Then this structure was encapsulated in polyimide tape (DuPont, Wilmington, DE) to shield the electrodes from air. The exposed ends of the Al foil from the electrode leads were strengthened with copper tape (Ted Pella, Redding, CA) to create a force sensor.

The sensors were subjected to ultrasonic waves at 1 MHz frequency to measure the piezoelectric outputs. An ultrasonic transducer manufactured by SoundCare Plus was used for this study. The Force sensor was taped to the benchtop to provide a hard supporting surface while an oscilloscope (PicoScope 4824, Pico Technology, UK) was connected to the reinforced electrodes to measure the open circuit voltage. The top of the sensor (the side facing up) was covered in ultrasound gel (purchased from Aquasonic). The output ultrasonic waves from the device were set at 1 MHz and 0.5 W/cm^2^. The 1cm^2^ transducer head was used to provide ultrasonic stimulation to the device (through the ultrasound gel) while the piezoelectric voltage was measured using the oscilloscope.

### Surface comparison of piezo PLLA films

By nature of fabrication, two surfaces of PLLA films possess contrast polarities: one side is more positive while the other side is more negative. To evaluate the more osteogenic favorable side, the pilot studies including alkaline phosphatase (ALP) and mineral deposition were conducted. As the results favored the negatively polarized surfaces, all of other studies were performed with cells seeded on the negative surfaces of our PLLA films. Film preparation and cell seeding followed the below procedures.

### Preparation of PLLA scaffolds for in vitro experiments

The scaffolds electrospun at 4000 rpm and cut at 45° were used as the experimental piezoelectric scaffolds whereas the scaffolds produced at 300 rpm and cut at 0° were used as non-piezoelectric controls.

The scaffolds were sterilized using a combination of ethanol and UV treatment under a laminar flow cell culture hood. First, the scaffolds were immersed in 70% ethanol for 30 minutes. Following that, the scaffolds were exposed to UV light for 20 minutes on each side.

After sterilization, the scaffolds were attached to the wells of a 6-well plate (purchased from Thermo Scientific) using a biocompatible silicone glue (KWIK-SIL produced by World Precision instruments). The bottom surface of the wells is covered with the silicone glue and the scaffolds were placed on the glued well surface before the silicone cured. The glue sticks the scaffolds securely on the plate during US treatments. Additionally, silicone prevents the ultrasound waves from getting reflected around the walls of the polystyrene wells and dissipate. Before seeding cells, scaffolds were washed 5 times with sterile PBS to remove any alcohol and silicone residues.

### Tissue culture

Human adipose derived stem cells (hADSCs) were purchased from ATCC at passage 1. The frozen cells received from the company were thawed, plated and expanded until the 4^th^ passage, as per the manufacturer’s protocol. For expanding the cultures, we used a growth media prepared using Dulbecco’s modified Eagle’s medium (DMEM), 2% fetal bovine serum (FBS) and 1% antibiotic solution pennstrep (containing 50% penicillin and 50% streptomycin) (all three components purchased from Gibco). For initial thawing, the frozen cell suspension was melted, and the cells were removed from the freeze media by centrifuging, pelleting and finally resuspending in fresh media before plating it into a flask of appropriate size. Expansion was carried out at 80–90% confluence. Finally, at passage 4, the cells were collected from the flasks using Trypsin / EDTA (Ethylenediaminetetraacetic acid) (obtained from Gibco) and seeded onto the PLLA scaffolds at a seeding density of 5 x 10^5^ cells. The cells were allowed to attach overnight in growth media. After that, the cells were transferred to osteogenic differentiation medium containing 50 μg/ml ascorbic acid and 10 mM beta-glycerophosphate added to the proliferation medium. The cultures were conditioned in osteogenic differentiation medium for 1 day before starting the daily ultrasonic treatments.

Mouse adipose derived stem cells (mADSCs) were purchased from iXCells Biotechnologies. The proliferation media used for these cells contained DMEM, 10% fetal bovine serum and 1% antibiotic pennstep solution. The rest of the procedures followed were the same as the hADSCs.

### Ultrasonic (US) treatment on the ADSCs

Ultrasound treatment at 40 kHz: A sonication bath (Branson 2800 CPX series) was used for this treatment which was carried out for 20 minutes each day. In order to prevent contamination of the cells by the bath water, the media in the wells was reduced (from 2 ml to 1 ml) to prevent spillage and the entire plate was sealed in a waterproof casing.

First, the lid was taped to the well plate. Next the plate is encapsulated in two layers of plastic wrap (Kirkland Signature Stretch-Tite Plastic Wrap—11 7/8 x750 Feet). Lastly, the plate was wrapped with duct tape (manufactures by 3M) such that the bottom of the plate has four layers of tape. To make the casing completely waterproof, a small piece of tape was added to each corner of the plate. A tab was created on the top of the encapsulation for suspending the plate into the water of the sonication bath. The plate was suspended in the ultrasonic bath using a laboratory clamp and stand setup so that it was submerged levelly and halfway in the water. After 20 minutes of sonication, the plate was removed from the bath and the encapsulation was removed with a pair of scissors. The media was refilled into each well and the plate was returned to the incubator until the next US treatment.

Ultrasound treatment at 1 MHz: An ultrasonic transducer (manufactured by SoundCare Plus) was used for this treatment with the duration being 20 minutes per day as in case of the 40 kHz treatment. The 5cm^2^ transducer head was used to stimulate each well individually at 1 MHz and 0.5 W/cm^2^. For this treatment, the lid was taped to the plate using labelling tape and the plate was suspended horizontally using a laboratory clamp and stand setup. Before treatment, the bottom of each well was covered in ultrasonic gel and the transducer head was set up such that it touched the bottom of the well to be treated and is parallel to the well bottom. After the treatment, the labelling tape was removed, and the plates were returned to the incubator until the next US treatment.

### Cell migration assays

For this experiment, a 6 well plate with transwell inserts (from Sigma Aldrich) was used. A PLLA scaffold was attached to the bottom of the well plate using silicone glue and hADSCs were seeded on top of the transwell mesh. In this study, we will assess migration by quantifying the number of cells that migrate from the top of the transwell mesh to the bottom side towards the piezoelectric charge of the scaffolds. The cells were allowed to attach overnight before filling the wells with media so that the media in the transwell floods into the main well. Then the plate is treated with ultrasound at 1 MHz for 20 minutes. Afterwards, the plate is returned to the incubator overnight to allow the cells to migrate. After 24 hours, the mesh was cut out of the transwell inserts for staining and imaging. At first, the cells that remained on the upper surface of the transwell membranes were washed with PBS and removed by using a cotton swab. The migrated cells that were attached on the underside of the filter membrane were fixed in cold 100% methanol for 15 minutes. Then, the membranes were stained using a 50 μg/ml DAPI solution (Sigma-Aldrich) for 10 minutes and mounted on cover slides. The migrated cells were imaged under a Fluorescent microscope (Leica) and quantified using ImageJ.

### Quantification of polarity based osteogenic activities

For the polarity studies, the osteogenic properties of negative (top) surface of the electrospun PLLA was compared against the positive (bottom) surface using mADSCs and ALP and Alizarin red assays. These results were normalized using the total protein content of the cultures quantified using the BCA assay (the Pierce™ BCA Protein Assay Kit purchased from Thermo Scientific). Protein extraction from the cells was performed by using RIPA buffer. The protocol from the manufacturers of the kit was followed to obtain the total protein quantification of each sample. The protein extracted was used for the ALP quantification as well as BCA assay.

For the ALP quantification, a pNPP (p-Nitrophenyl Phosphate) based assay purchased from Bio-Rad was used. The standards were prepared by dissolving different amounts of ALP (purchased from Sigma-Aldrich) in an aqueous solution. The pNPP solution was prepared as per the kit protocol and added to the protein extracts and the standards. The ALP levels in the samples and the standards were quantified in accordance with the protocol of the kit.

For the Alizarin red assay, the cells on the scaffolds were fixed in 70% ethanol at 4°C for one hour. Once fixed, the Alizarin red dye (purchased from EMD Millipore corp.) was added to the wells, and they were incubated at room temperature for 30 minutes. Then, the dye was removed, the wells were rinsed and then viewed macroscopically and under the microscope to visualize the mineral formation (stained in red). The amount of mineral formed is quantified by dissolving the alizarin red dye in the culture plate wells in cetylpyridinium chloride (purchased from Sigma Aldrich) and measuring the absorbance of the solutions.

### BMP-2 and TGF-β quantification

Both BMP-2 and TGF-β levels were quantified by Elisa. BMP-2 (Catalog number DBP200) and TGF-β (DB100C) ELISA kits were purchased from R&D Systems. First, the films were cut (1.27 cm x 1.27 cm), sterilized and attached to 6-well plates. Next, human ADSCs were seeded on PLLA films as described above. Similarly, cells seeded on tissue culture plates with osteogenic differentiation medium served as control groups while the same cells in normal growth medium served as reference groups. Next, cells underwent daily ultrasound treatments for 4 days to progress the osteogenesis. Right after the last treatment, cells media were replaced with incomplete media (basal DMEM without FBS containing 50 μg/ml ascorbic acid and 10 mM beta-glycerophosphate; reference groups were added with basal DMEM only). As cells were incubated overnight, supernatants were collected to quantified secreted growth factors. BMP-2 and TGF-β ELISA assays were performed per manufacturer’s protocol. Meanwhile, relative cells density was quantified using Pierce BCA protein assay kits (PI23227, Thermo Scientific). Cells were washed with cold PBS before being lysed with a prepared cell lysis buffer (RIPA, Catalog R0278, purchased from Sigma-Aldrich, added with Protease and Phosphatase Inhibitor cocktail, Catalog PPC1010, purchased from Sigma-Aldrich, at 1% v/v). For each well, 500 μL lysis buffer was added, incubated 10 minutes in fridge before supernatants were collected to perform further studies.

### Live calcium ion imaging

Live calcium ion staining and imaging was carried out to visualize the increase in intracellular calcium signaling in the cells as they get exposed to the piezoelectric surface charges of the PLLA films under US. For this assay, hADSCs were seeded on the films and allowed to attach overnight. Following this, the cells were stained with a Cal-520 AM dye (from AbCam) to visualize the calcium ion concentration in the culture. After the staining was completed following the manufacturer’s protocol for the dye, the films were transferred to glass bottom plates for imaging. Imaging was done using a fluorescent microscope (Leica) and each sample was imaged while being treated with US as well as without any US treatment to visualize the changes in calcium ion concentration. These changes were then quantified using ImageJ.

### Intracellular calcium signaling assay

Calcium colorimetric assay (Catalog MAK022) was purchased from Sigma-Aldrich. Similar to previous studies, human ADSCs were seeded on our piezo- and non-piezo films, contrasting to cells seeding on tissue culture plates. Cells grew in respective media and underwent daily ultrasound treatments for one week. Next, media were removed, and cells were washed with Dulbecco’s PBS before lysed with 1ml of 2% v/v Triton X-100. Lysates were then quantified for intracellular calcium following manufacturer’s protocol. Meanwhile, relative cell numbers were quantified with Quantiflour dsDNA system (Catalog E2671, Promega).

## Results

### Piezoelectric property evaluation

**Fig 2:**
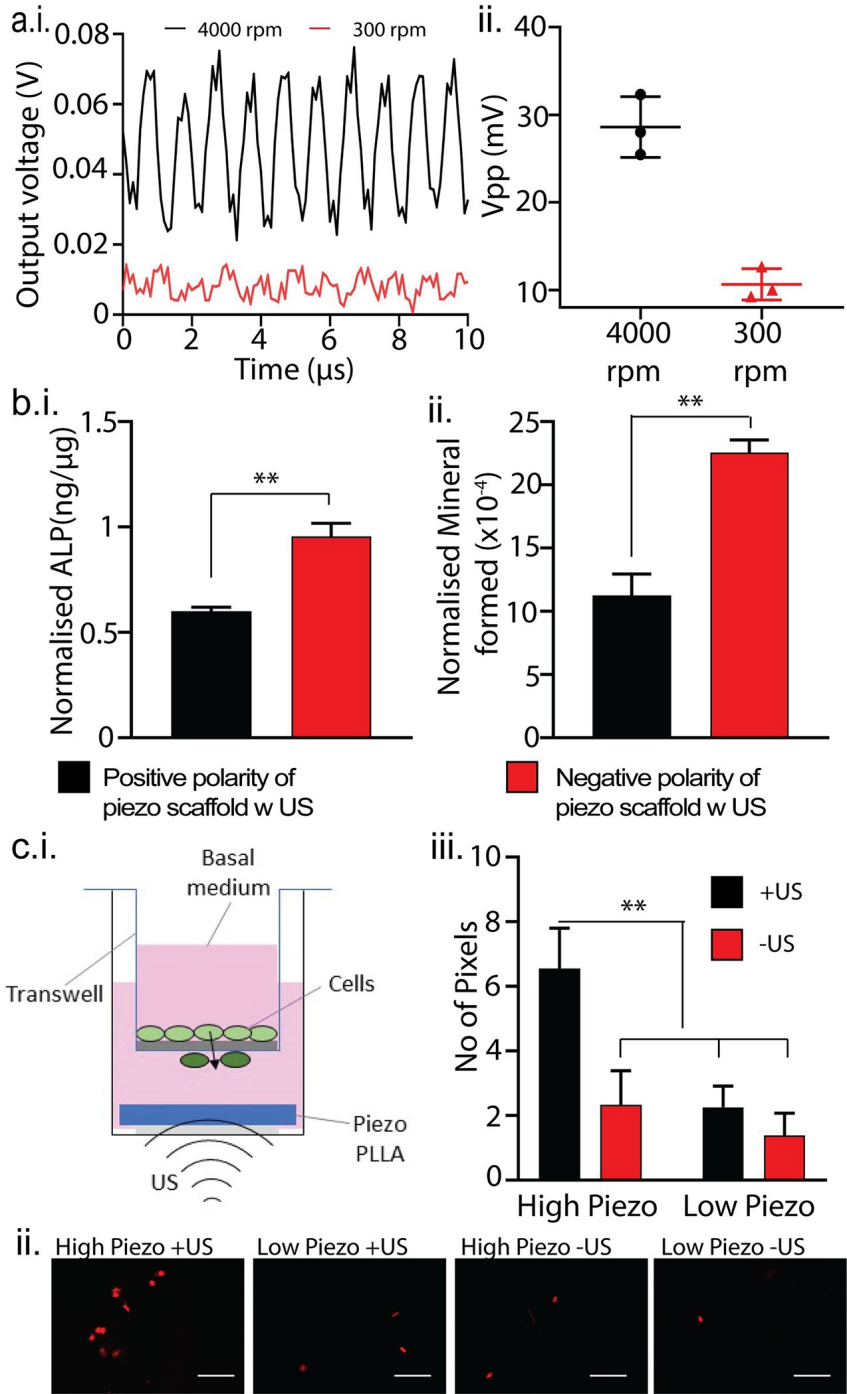
Biophysical characterization of the piezoelectric PLLA scaffold. **a.** Open circuit output voltage from the PLLA sensors made with films fabricated at 4000, and 300 rpm collector speeds under 1MHz and 0.5 W/cm^2^ ultrasound represented as **i.** waveforms and **ii.** peak output voltages. **b.** A quantitative comparison of the osteogenic properties of the positive and negative polarity sides of the piezoelectric scaffold using **i.** alkaline phosphatase production and **ii.** Mineral formation using alizarin red. (n = 3 samples each group). **c.i.** Simplified schematic showing the setup used for the cell migration experiment using a transwell well plate setup. **ii.** Representative fluorescent images of cells migrated onto the underside of the transwell membrane after being subjected to different treatments. All the scale bars are 50 μm. **iii.** Quantitative comparisons of the fluorescence expressions of the different groups with and without US (n = 3 transwell membranes for each group). A student T test was used to calculate statistical significance for figure **b** whereas one-way analysis of variance (ANOVA) followed by Tukey’s post hoc test was used to calculate statistical significance for figure **c**. In the figure, ** represents a significance level of 0.01.

Electrospinning was used to fabricate the piezoelectric PLLA nanofiber mats and followed by a two-step annealing process and cutting the scaffold pieces at 45° to maximize the piezoelectric properties of the PLLA nanofibers as described in our previous report [11, 12, 21, 22, 24, 25]. As seen in **Fig 2A** and our reported works [11, 12, 21, 22, 24, 25], increasing the rotation speed of the drum increased the fiber and crystal alignment which leads to higher piezoelectric outputs of the materials. The fibers produced at 4000 rpm had significantly higher output voltages than the ones produced at 300 rpm under the same applied US [22]. Hence, the 300-rpm films were used as the negative control and the 4000 rpm films as the piezoelectric experimental groups for the rest of the studies. For convention, we refer to the 300 rpm films as low piezoelectric samples and the 4000 rpm films as the high piezoelectric samples. We have reported other material properties of the electrospun PLLA scaffolds like fiber alignment, chemical composition and crystallinity in our previous works [11, 12, 21, 22, 24–26].

### Osteogenic studies under different polarities

The osteogenic effects of different surface charges of different polarities were studied. When the films were placed under any form of mechanical stress, the side of the film that was attached to the collector drum during electrospinning developed a negative charge whereas the other surface develops a positive charge [12]. mADSCs were seeded on each side of piezoelectric scaffolds and treated with US to induce different surface charges on both sides. The ALP quantification (**Fig 2.B.i**) and Alizarin red staining (**Fig 2.B.ii**) results showed that cells on negative side demonstrated higher osteogenic activities than those on the positive side. This osteogenic nature of negative surface charges has also been reported in other studies [12, 27]. Therefore, the negative side of the scaffolds is used for the following studies, except for the experiments comparing the two different polarities.

### Cell migration

The ability of the piezoelectric charge of our films to induce cell migration was then studied using a transwell setup [28, 29]. In this experiment, the cells were seeded on top of the transwell membrane while the PLLA scaffolds were placed in the receiving well (**Fig 2.C.i**). As seen in the representative images in **Fig 2.C.ii.,** the cells exposed to the high piezoelectric scaffolds with US treatment had a much higher number of migrating cells visible on the underside of the transwell as compared to the controls. The extent of cell migrations was quantified using ImageJ (**Fig 2.C.iii**). The results showed that the high piezoelectric scaffolds under US facilitated a significantly higher amount of cell migration than the controls. This indicates that the surface charge generated in the piezo scaffolds by the US stimulation is the driving force for the cell migration.

### Cytokine production

**Fig 3:**
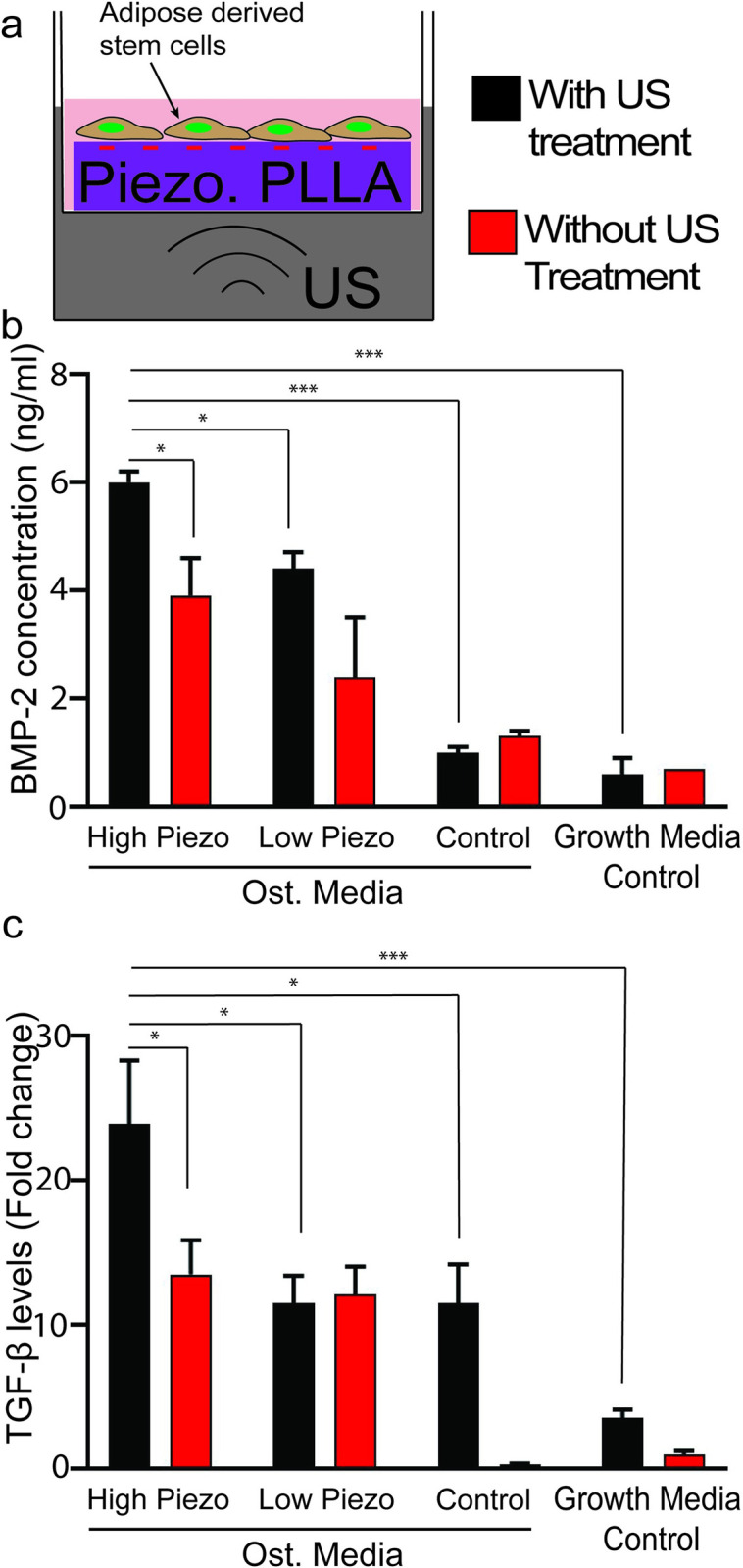
Cytokine expression by ADSCs, grown on the piezoelectric PLLA nanofiber scaffold under applied US *in vitro*. **a.** A schematic representation of our setup for seeding ADCSs onto the negative side of the PLLA scaffolds and treating them with US. The US generates a controllable pressure, inducing the formation of electrical charges on the surface of the nanofiber films, which facilitate osteogenesis of the cultured cells. **b.** A quantitative comparison of BMP-2 produced by the cells in different treatment groups. **c.** A quantitative comparison of TGF-β produced by the cells in different treatment groups. All data were normalized with relative cell population quantified in protein assays (n = 3). One-way ANOVA followed by Tukey’s post hoc test was used to calculate statistical significance. In the figure, * represents a significance level of 0.05, and *** represents a significance level of 0.001.

Next, we carried out *in vitro* experiments to identify the cytokines which are expressed by the ADSCs and help them to differentiate into the osteogenic lineage. The schematic in **Fig 3A** describes the setup of our in vitro experiment in which we apply US (1 MHz, 0.5 W/cm^2^, 20 minutes/day) on different PLLA films, seeded with ADSCs (see methods in experimental section). Supernatants produced by the cells were used to quantify the amounts of Bone morphogenic protein-2 (BMP-2) and transforming growth factor-β (TGF-β) released during osteogenesis [30–33]. The results (**Fig 3B** and **3C**) showed that the expressions of both cytokines were significantly higher in the cells seeded on the high piezoelectric scaffolds undergoing US treatment compared to the other control and sham groups in the same condition of using osteogenic medium and growth medium.

### Intracellular calcium signaling assessment

Lastly, we determined the ability of the piezoelectric surface charges to induce intracellular calcium signaling using a live calcium imaging assay and an intracellular calcium signaling quantification kit.

**Fig 4:**
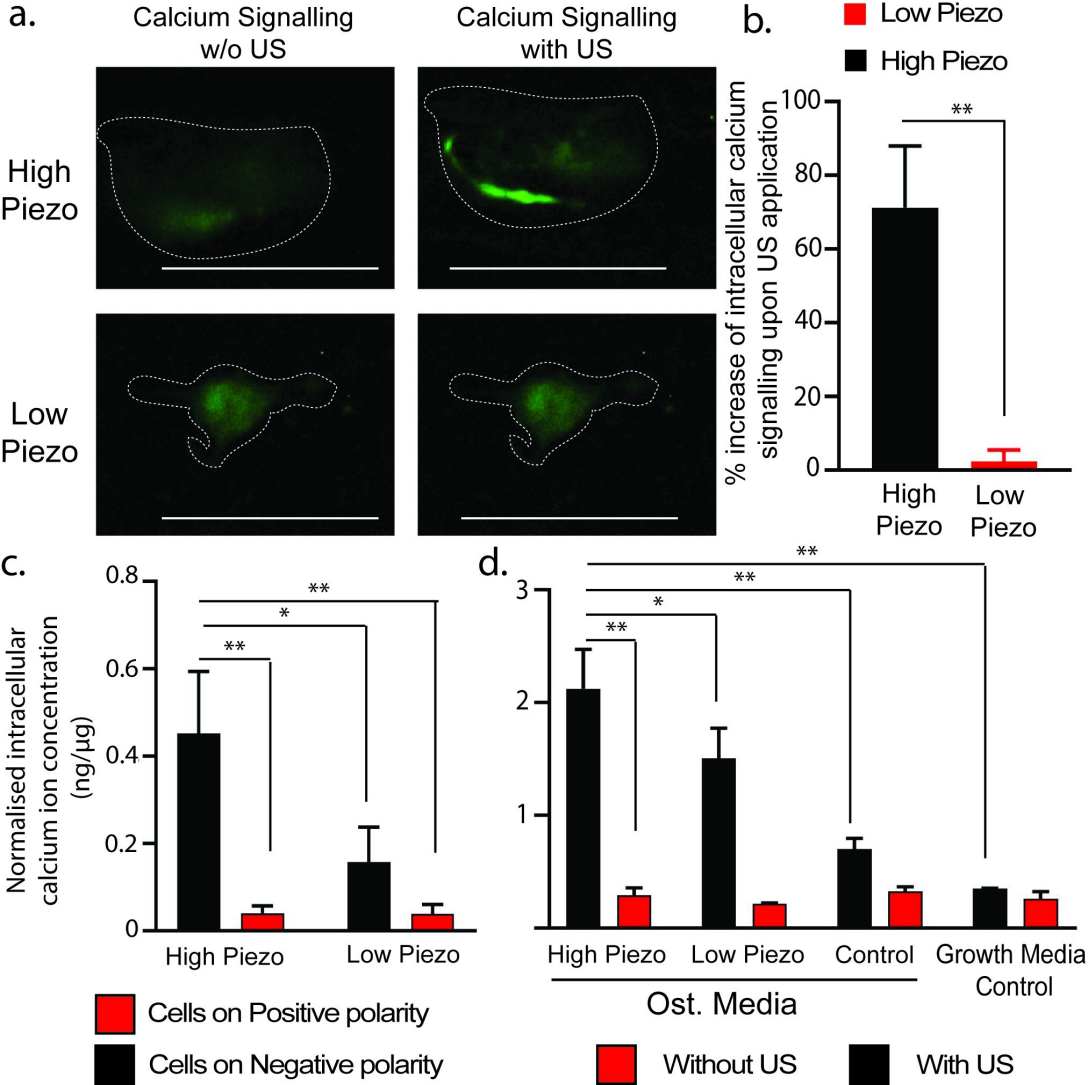
Calcium signaling activity of ADSCs when grown on the electrospun PLLA scaffolds with US treatment. **a.** Representative fluorescent images of the cells seeded on different scaffolds with and without US treatment as observed under the green channel (to observe the calcium signaling). All the scale bars are 10 μm. **b.** A graphical comparison of the increase in fluorescent expressions (observed under the green channel representing calcium signaling) upon application of US (n = 3 for each group). The fluorescence levels in the absence of US treatment are used as baseline. **c.** A quantitative comparison of the intracellular calcium signaling in cells seeded on the positive and negative sides of the piezoelectric and non-piezoelectric scaffold under ultrasound. **d.** A quantitative comparison of the intracellular calcium signaling in cells exposed to different treatments. All data were normalized with relative cell population quantified in DNA assays (n = 3). A student T test was used to calculate statistical significance for figure **b** whereas one-way ANOVA followed by Tukey’s post hoc test was used to calculate statistical significance for figures **c** and **d**. In the figure, * represents a significance level of 0.05, and ** represents a significance level of 0.01.

Live Calcium images were observed and analyzed closely for individual cells. As seen in the representative images in **Fig 4A**, the cell seeded on the high piezo scaffold demonstrates a significant increase in intracellular calcium concentration (as seen by the increase in green fluorescence) as compared to the non-piezo controls. The increase in cell fluorescence is quantified using ImageJ (**Fig 4B**). These results confirm that the US treatment on the high piezo scaffold facilitates a significant increase in cell fluorescence and hence the intracellular calcium concentration as compared to the control. For the intracellular calcium ion quantification assay, we performed two different studies. Firstly, we determined if different surface charge polarities demonstrated different levels of intracellular calcium signaling which are normalized to total DNA concentration. For this, we seeded ADSCs on both the positive and negative sides of our scaffolds and stimulated them with US (of 1 MHZ frequency and 0.5 W/cm^2^ intensity). Our results (**Fig 4C**) showed that the cells seeded on the negative side of the high piezoelectric samples (fabricated at 4000 rpm) treated with US had significantly higher intracellular calcium levels as compared to the cells seeded on the positive side or on the low piezoelectric samples (fabricated at 300 rpm). These results are consistent with the results in **Fig 2C**. Additionally, we also compared the intracellular calcium levels in cells seeded on the different scaffolds (on the negative sides) as well as tissue culture plates with and without US (**Fig 4D**), again normalized to the total DNA content. The results showed that the cells seeded on the high piezoelectric scaffolds with US treatments had significantly higher intracellular calcium ion levels as compared to all the other controls.

## Discussion

Piezoelectric materials like PLLA can generate electric charges when subjected to mechanical deformation. The interplay between mechanical and electrical stimulation in such materials is important and their combined effects have been thought to influence cellular behaviors like proliferation, migration, and differentiation. The present study unveils a novel observation regarding the unique role of piezoelectrical charges in influencing cellular behavior. In this study, we show that the inherent osteogenic properties of PLLA based piezoelectric stimulation operates through increased intracellular signaling (resulting from the piezoelectric charges) which in turn upregulates the production and release of cellular osteoinductive growth factors such as BMPs and TGF-beta) (**Fig 1**). We selected the ADSCs as the cell model because the cells have been shown to exhibit osteogenesis, equivalent to the bone marrow stem cells (BMSCs) while they are also easier to be collected and expanded for extensive *in vitro* experiment. Regardless of the stem cell source, as long as we use the same condition for all experiment and control groups including non-piezoelectric or no-US for comparisons, our experiment will validate the comparison of outcomes between the groups. We studied how different surface charge polarities could have different effects at the cellular levels and affect osteogenic differentiation. Our results demonstrated that a negative surface charge has higher osteogenic properties as compared to positive surface charge, which has already been confirmed by existing studies [12, 27]. We have demonstrated that piezoelectric charges generated on PLLA materials under mechanical activation using ultrasound augment stem cell migration (**Fig 2**) which has also been confirmed by existing literature [34, 35].

Our results also show that the negative piezoelectric charge produced by the PLLA under US upregulated production of cytokines like BMP-2 and TGF-β (**Fig 3**). Previous studies have shown that individually, US treatments and negative surface charges are conducive to BMP-2 production in cells [12, 36]. Similar results have been reported for TGF-β release from cells [37–39]. Hence, our results make it clear that negative piezoelectric charges that are induced in our scaffolds by the US treatments is able to upregulate production of valuable cytokines like BMP-2 and TGF-β that play an important role in facilitating osteogenesis in the seeded cells. One thing to note is that the piezoelectric stimulation in a normal medium is not able to induce osteogenesis. On the other words, the piezoelectric stimulation merely acts to enhance the osteogenesis but cannot induce osteogenesis on its own without the presence of osteogenic differentiation media (ODM) or some other osteoinductive material. We believe that the *in vivo* bone injury in nature would already make the body secrete such the biological signals and therefore, the piezoelectric stimulation would be still beneficial for bone healing without such an exogenous signal (like the ODM) as shown in our previous *in vivo* result [40]. Lastly, we demonstrate that negative piezoelectric charges lead to a greater upsurge of intracellular calcium signaling (**Fig 4**). Calcium signaling plays a very crucial role in osteogenesis, bone remodeling and matrix formation [41, 42]. Previous studies have shown that both negative charges and mechanotransduction (caused by mechanical stimulations like US) can enhance intracellular calcium signaling individually [43, 44]. The generated negative piezoelectric charges could directly trigger the differentiation of stem cells into bone cells, via opening of calcium channels and intracellular calcium signaling. Our results also indicate that cells seeded on the high piezoelectric scaffolds with US treatment have a significantly higher intracellular calcium concentration compared to controls like non-piezoelectric scaffolds or tissue culture pates with or without US. Thus, the negative piezoelectric surface charges induced by the US enhance intracellular calcium signaling much more significantly than US treatment alone. This calcium signaling is responsible for upregulation of valuable cytokines like BMP-2 and TGF-β which ultimately results in the induction of osteogenesis in the cells seeded on the materials [5, 45, 46].

The osteogenic effects observed in high piezoelectric PLLA scaffolds (which in this study, provides a combination of electrical stimulation and mechanical stimulation by US) seems to be likely due to electrical stimulation more than mechanical stimulation alone. This can be seen in our comparison of the low piezo groups with and without US which don’t show the significant differences as the high piezo + US group. These results highlight the importance of understanding the role of electrical fields and surface charge on cellular behavior, especially in the context of stem cell differentiation and tissue engineering [12, 22]. This work helps us to understand the cellular mechanisms behind the osteogenic nature of piezoelectric charges and opens up new possibilities in terms of utilizing and optimizing piezoelectric biomaterials like PLLA (with varying piezoelectric strengths and stimulations) to engineer bone and other tissue.

## Conclusions

Here we present a mechanistic study that attempts to understand the osteogenic properties of the piezoelectric PLLA scaffold that we presented previously [12, 22]. In our previous works, we have fabricated PLLA bone scaffolds using a technique that maximizes its piezoelectric output and activating the piezoelectric properties using external US for bone regeneration. In this work, we studied the cellular mechanisms behind the osteogenic regeneration capabilities of this technology. Our results have shown that 1) Piezoelectric charges generated in the PLLA scaffolds under US enhance cell migration and intracellular calcium signaling in stem cells (using ADSCs as the cell model), 2) Negative charges have a stronger osteogenic effect on the ADSCs as compared to positive charges because negative charges are more effective in opening calcium channels and increasing intracellular calcium ion oscillations, 3) This increased intracellular signaling upregulates production and release of key cytokines like BMPs and TGfs that play a crucial role in cellular osteogenic differentiation.

This study will help us understand the mechanisms behind the osteogenic nature of the negative piezoelectric charges in our PLLA scaffolds. However, further studies are required to optimize the regenerative capabilities of this technology. Firstly, the optimal amount of piezoelectric charges and US intensity for osteogenesis will need to be investigated by tailoring the applied US and/or the piezoelectric effect of the PLLA nanofiber film. Secondly, the effects of the piezoelectric charge on other vital cytokines like Insulin like growth factors (IGFs), osteorix etc. need to be studied. Finally, the roles of other types of cells (other than bone cells) like macrophages and endothelial cells in bone regeneration *in vitro* and *in vivo* needs to be investigated further in order to understand the full picture of how piezoelectric charges can facilitate regeneration of tissues like bone. We plan to perform these studies in the next phase of this work which will demonstrate the understanding and optimization of the osteogenic effect of this novel biodegradable PLLA scaffold that acts as a battery-less and remotely controlled electrical stimulator.

## Supporting information

S1 Data (ZIP)
