# Supplementary figures and images for: Osteo-inductive effect of piezoelectric stimulation from the poly(l-lactic acid) scaffolds
Source: PLoS One. 2024 Feb 27;19(2):e0299579. doi: 10.1371/journal.pone.0299579 (PMC10898771; doi:10.1371/journal.pone.0299579)

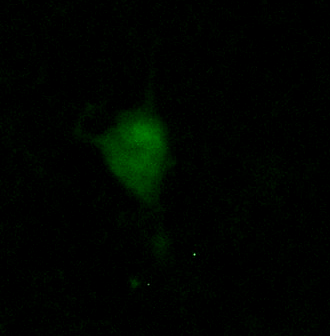

Supplement: S1 Data — (ZIP) [file pone.0299579.s001.zip › Data/Calcium ion signalling image analysis/300+US green channel cropped 2.tif]

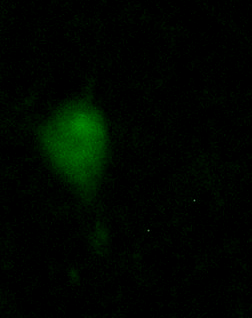

Supplement: S1 Data — (ZIP) [file pone.0299579.s001.zip › Data/Calcium ion signalling image analysis/300-US green channel cropped 2.tif]

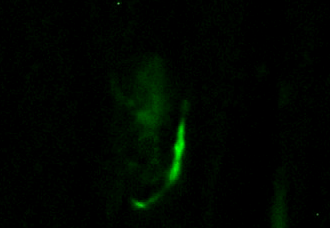

Supplement: S1 Data — (ZIP) [file pone.0299579.s001.zip › Data/Calcium ion signalling image analysis/4k+US green channel cropped 1.tif]

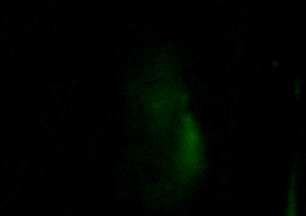

Supplement: S1 Data — (ZIP) [file pone.0299579.s001.zip › Data/Calcium ion signalling image analysis/4k-US green channel cropped 1.tif]

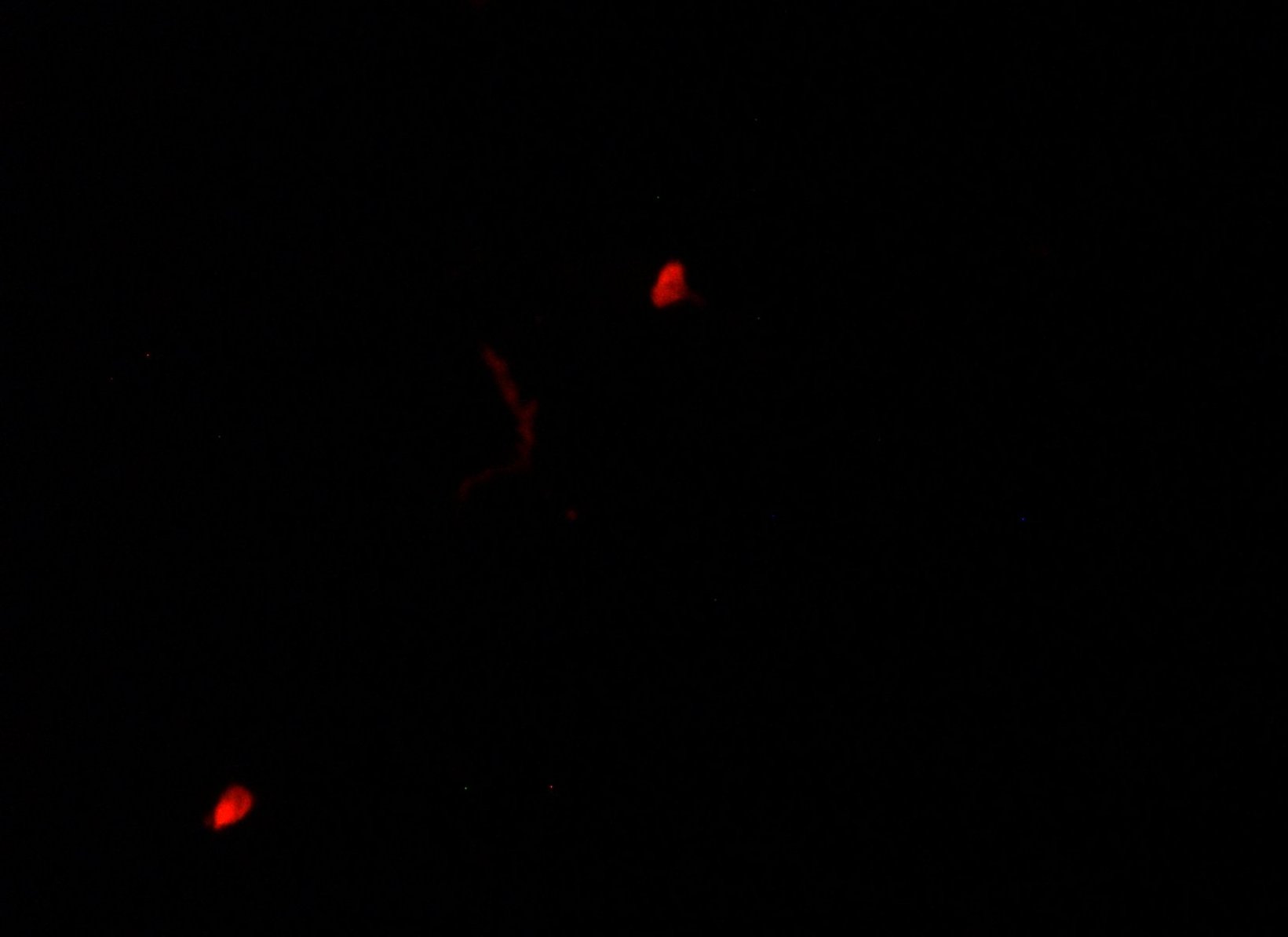

Supplement: S1 Data — (ZIP) [file pone.0299579.s001.zip › Data/Cell migration images (transewll)/High Piezo.jpg]

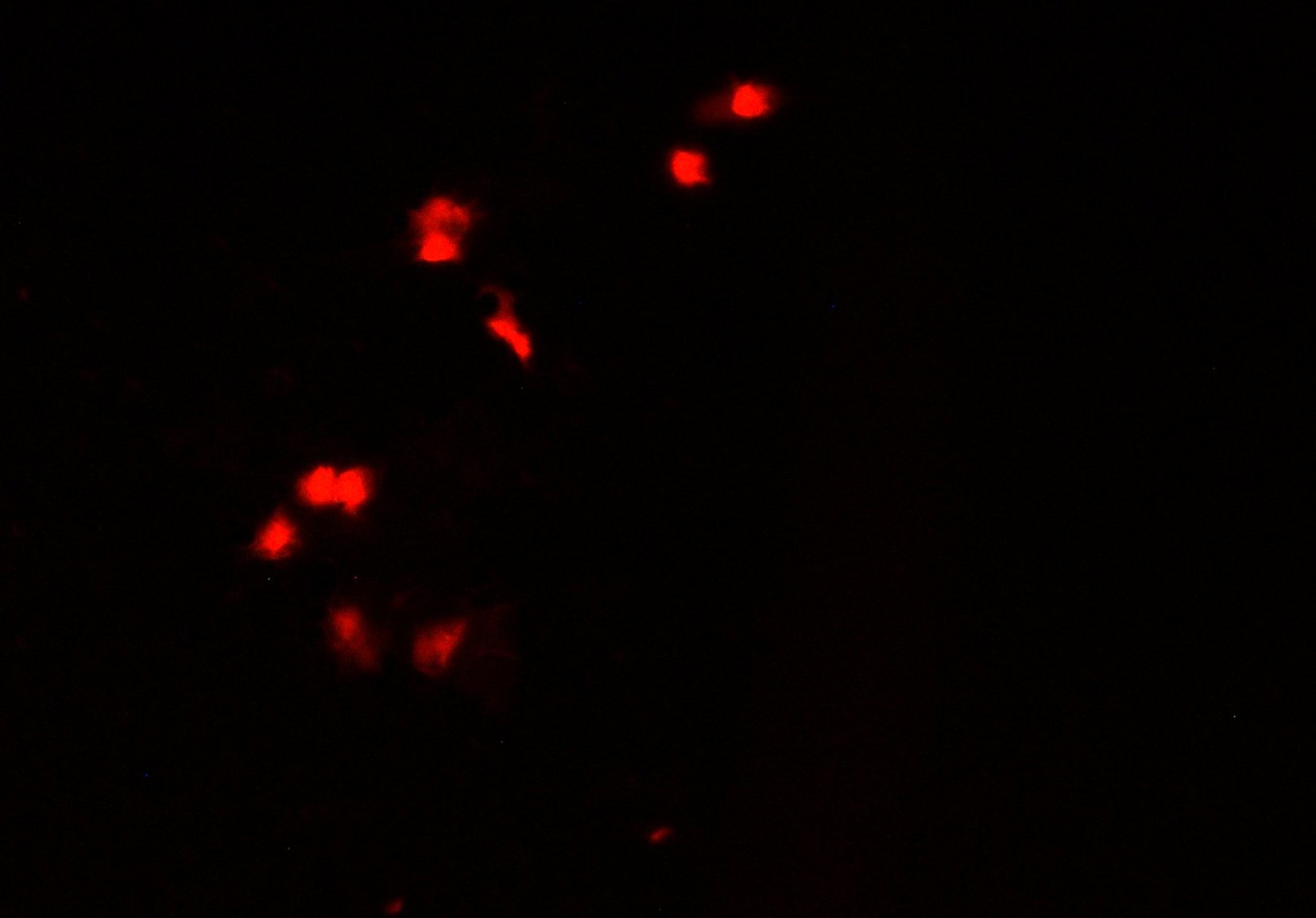

Supplement: S1 Data — (ZIP) [file pone.0299579.s001.zip › Data/Cell migration images (transewll)/High piezo+US.jpg]

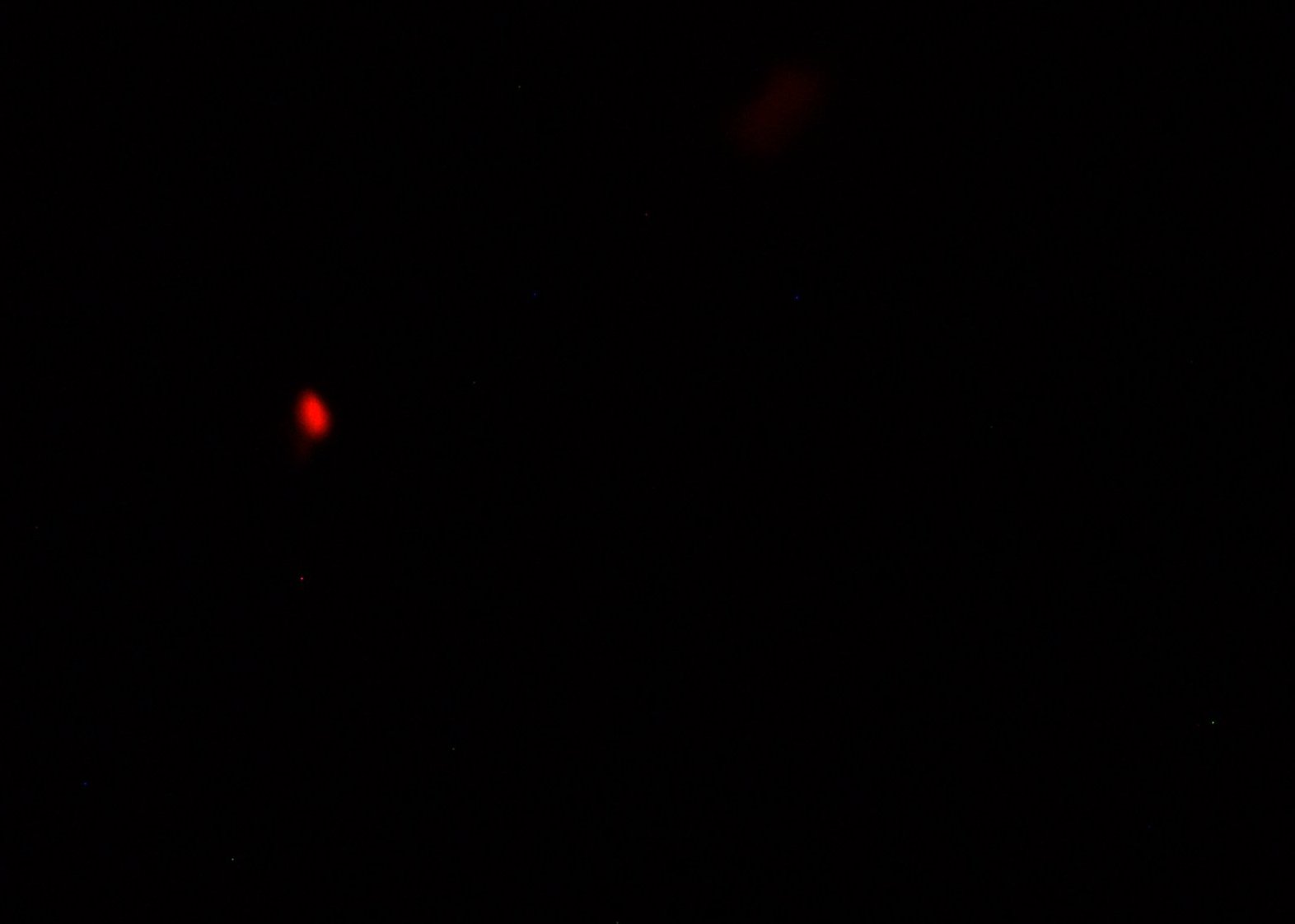

Supplement: S1 Data — (ZIP) [file pone.0299579.s001.zip › Data/Cell migration images (transewll)/Low piezo.jpg]

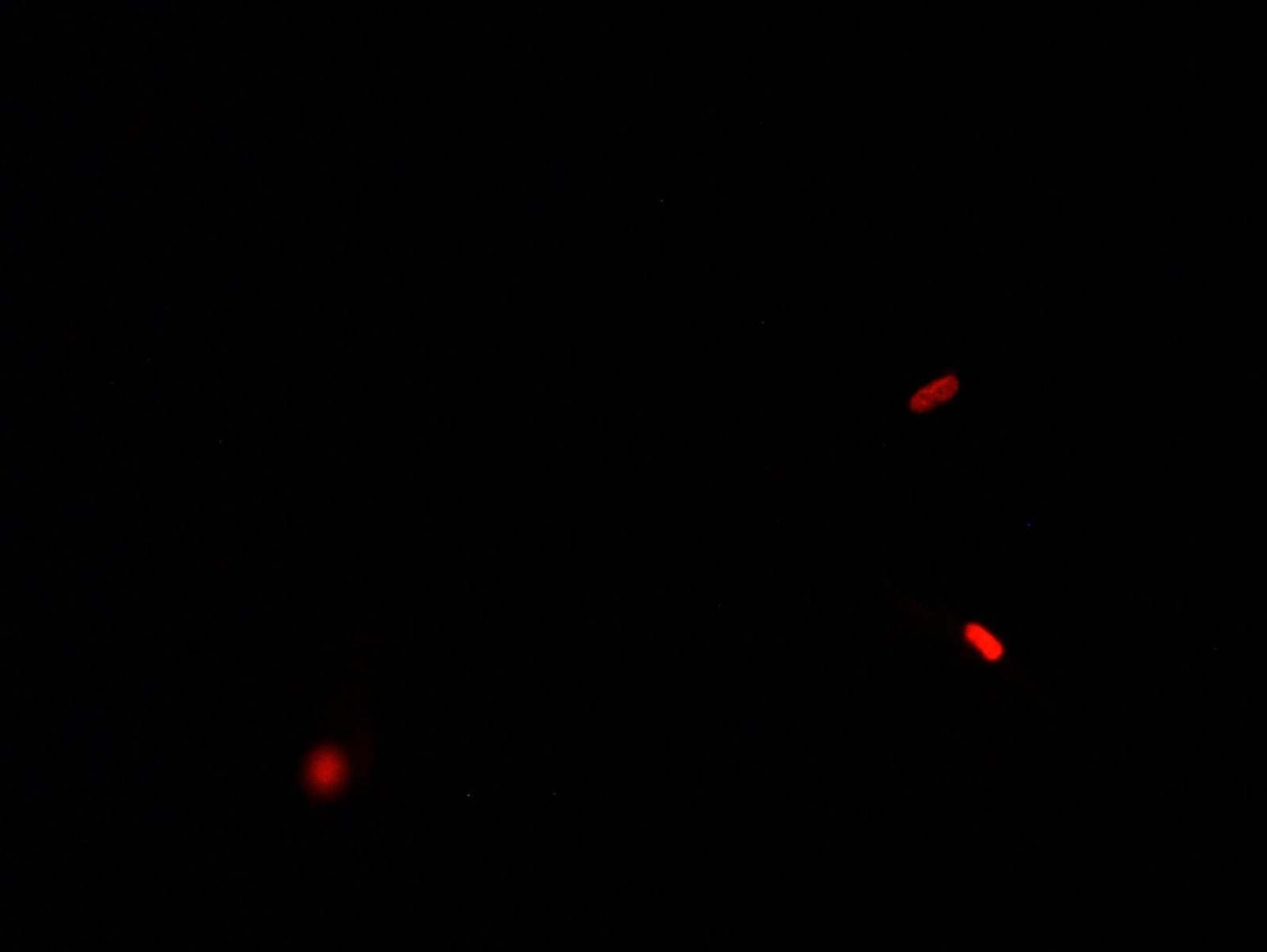

Supplement: S1 Data — (ZIP) [file pone.0299579.s001.zip › Data/Cell migration images (transewll)/Low piezo+US.jpg]
